# Hospitals' contributions to their communities: Should they be regulated?

**DOI:** 10.3389/fpubh.2022.1064210

**Published:** 2022-11-24

**Authors:** Cory E. Cronin, Simone R. Singh, Jason S. Turner, Connie J. Evashwick

**Affiliations:** ^1^Department of Social and Public Health, College of Health Sciences and Professions and Appalachian Institute to Advance Health Equity Science, Ohio University, Athens, OH, United States; ^2^Department of Health Management and Policy, University of Michigan, Ann Arbor, MI, United States; ^3^Department of Health Services Management, Rush University, Chicago, IL, United States; ^4^Milken Institute School of Public Health, George Washington University, Washington, DC, United States

**Keywords:** tax-exempt hospitals, not-for-profit hospitals, hospitals & community, hospital community benefit, community benefit regulation, anchor institutions

## Introduction

Hospitals are often epicenters of their communities. In addition to the role they play in providing clinical care for the ill and supporting population health efforts, many hospitals have a mission that includes caring for all members of the community, especially those in need. Recent efforts have been made by governments to set expectations for how hospitals contribute to their community beyond the provision of patient care. This has been codified into law in the United States of America (USA) over the past two decades (1), but considered and discussed in other countries as well (2, 3). We raise the question here: *To what extent is it necessary or even beneficial to regulate hospital-community relationships through public policy?*

We propose the following inquiries.

- First, does a hospital contribute to its community, beyond the acute care services it offers, naturally and organically, or must the role of the hospital in the community be mandated by legislation and regulation?
- Second, if regulated, what constitutes a “contribution” to the community's health and wellbeing, and can that contribution be measured?
- Third, can the impact of the hospital's activities on the community's health status be measured? If so, what metrics should be used, and what is the target, standard, or threshold hospitals must reach to fulfill their community benefit obligations?

We consider each of these questions briefly, recognizing that more extensive analyses are warranted.

## Hospitals' contributions to their communities

Hospitals now and throughout history have been social institutions and have offered myriad services to their constituents. Numerous examples exist, from Hotel Dieu in Paris to the almshouses of medieval Europe and the nascent USA, to the early seamen's hospitals that isolated sailors with contagious diseases to prevent the spread of infections in the community (4–7). Hospitals throughout the world founded by religious institutions have served as providers of health care but also as schools and community centers. Today, the size and stability of many hospital organizations mean that their economic contributions as employers and purchasers of local services are so substantial that they are often considered to be critical anchor institutions responsible for improving the social determinants of health and community wellbeing through targeted hiring practices, selection of local vendors, career laddering, real estate development, and financial investment (8–10). Community outreach and engagement are prevalent in many forms, from organizing health fairs to allowing community support groups to use hospital space for meetings. Leaders of hospitals often report such activity as integral to their organizational identity and culture (11–13). The International Hospital Federation, soon to celebrate its 100th anniversary, documents how hospitals throughout the world have contributed to their communities in myriad ways. As an example, the newly formed Geneva Sustainability Centre (14) showcases current innovations by hospitals across the globe to protect the climate and environment.

## Regulation of community benefit in the USA

In the USA, government efforts to establish community expectations for hospitals have attempted to formalize hospitals' philanthropic foundations in order to capture tax revenues and, more recently, to address gaps in the healthcare delivery system. This effort initially dates back to a 1969 tax ruling, when the federal government first articulated expectations of “charity care” in exchange for exemption from federal income tax (15). About half of the 50 states comprising the USA have also enacted some form of regulation attaching exemption from state income tax to the provision of uncompensated care for those unable to afford care (16). The rationale is that not-for-profit hospitals are providing resources directly to the community in lieu of paying taxes. In 2022, 58% of community hospitals, or 2,960 in total, had not-for-profit status (17).

The USA federal tax authority, the Internal Revenue Service (IRS), requires that every organization that seeks to qualify for tax exemption must report annually on its activities and financial transactions. Not-for-profit hospitals' reporting of specific activities assumed to benefit the community began in 2009. Table 1 provides an abbreviated list of the types of activities USA hospitals report to the IRS and examples of what is reported.

**Table 1:** Hospital community benefit activities.

**Type of activity**	**Example**
Health promotion	Health fairs
	Providing vaccinations and testing
Health education	Holding classes for community members
	Outreach teaching to schools and community groups
Community building	Community gardens to grow produce
	Offering space for community meetings
	Hosting volunteers for community projects
Health professions education	Educating health professionals as field sites for training
	Collaborating with local schools for pipeline programs
Housing	Building/sponsoring low-income housing
	Supporting shelters with supplies or health care
	Modeling green buildings
Community economy	Hiring locals residents
	Buying locally produced supplies
	Buying locally produced foods
	Contracting with local services, such as laundry
Transportation	Running bus/van services
	Providing taxi vouchers for low-income patients
Food	Opening cafeterias to the community
	Giving excess food to community organizations
Research	Clinical or management research
Specialty high-cost	Emergency rooms
services	Neonatal care ICUs
	Burn units

The IRS regulations require extremely detailed and complex reporting, applying data from multiple departments in the hospital. There are now more than a dozen years of data to assess the impact of the community benefit policy. Although reporting has been somewhat codified by the information systems available, no metrics have been specified for each of the activities. For example, for health fairs, is reporting the number of people who come by a booth acceptable? Are denominators as well as numerators required? Each hospital defines its own “community” and can determine its own system of accounting for community benefit activities.

For most hospitals, the expectations are vague, both in regard to the efforts to be made and the extent of resources to be dedicated. In 2020, the General Accounting Office (GAO), a federal government agency, issued a report noting a lack of a “well-documented process” for the review of hospitals' community benefit activities (18). Six recommendations were offered to improve the community benefit reporting and monitoring processes. Whether or not these are being implemented is not evident as of 2022.

Many of the activities that count as community benefits are difficult to document, difficult to quantify, and difficult to assess. The most easily measurable aspect, and the one still most prominent in spite of conceptual shifts, is the cost of medical care provided at no or reduced cost to individual patients. The vast majority of USA hospitals' community benefit spending is focused on providing care to individual patients rather than dedicated to improving community health outcomes. Studies over the past ten or more years have consistently indicated that 85–90% of community benefit dollars reported by not-for-profit hospitals were attributed to uncompensated care (18–20). This proportion has prevailed despite the major financial changes brought about by the 2010 Affordable Care and Patient Protection Act (ACA) law, federal initiatives to promote value-based payment, the pandemic, or any other systemic change.

A final consideration is the cost to not-for-profit hospitals of conducting and reporting on community benefit activities. The costs of operating a department in a hospital that carries out community benefit activities or conducts the required 3-year community health needs assessment can be claimed as “community benefit.” These costs aren't thoroughly reported, and no national data exist. If one assumes that each of the 2,960 hospitals spends at least $100,000 annually, the annual cost to comply with the federal regulation is nearly $3 billion. Could this money be spent on community health in better ways?

## How much is “enough”?

Not-for-profit hospitals in the USA spent more than $100 billion on community benefit in 2017, according to pre-pandemic reporting (21). Although some states have specified a percent of gross or net patient revenues as a required minimum for state income tax exemption for hospitals, no financial target has been declared at the federal level. The federal government has no answer to the question asked by the hospital industry, “How much is enough?”

At the same time, proprietary (for-profit) hospitals in the USA have been compared to not-for-profit hospitals with regard to their contributions to the community. A number of studies have found little difference in types of activities or estimated dollar amounts (12, 13, 22). Government and select specialty hospitals are exempt from the community benefit requirement. If the assumption is that the hospital is a social institution with an obligation or mission to serve its community, why should the government excuse its own institutions from making similar contributions?

What happens when a not-for-profit hospital experiences a deficit? Is it expected to withdraw from the community, or to continue with community outreach at the expense of acute inpatient care? The law in the USA is silent.

A major flaw of the community benefit policy is that no logic model delineates how any of the activities that “count” toward community benefit relates to the overall health of the community. Despite the array of data sets that characterize the health behaviors or health status of a community, no single measure of a “healthy community” has been determined. Moreover, the methods for measuring impact of any single intervention on a community characteristic are not fully refined. We can say that less smoking is better than more smoking for one's health, and some techniques are effective at reducing smoking in some populations, but we can't precisely measure the impact on the health of the community of a community-wide stop-smoking campaign organized by a hospital but supported by many institutions throughout the community. The question of attribution is neither raised nor addressed in the reporting methodology. A recent collection of research articles pertaining to community benefit provided scarce evidence that the policy at the federal or state level has a direct and measurable change on the health status of a community (23). Are hospitals the most appropriate mechanism to fill gaps left by deficiencies in the health system?

## Discussion and conclusions

Of the three questions posed initially, we conclude: (1) Hospitals of all types throughout the world contribute to their communities in myriad ways without laws that require them to do so. Mission is often a stronger driver than regulations. (2) The many ways in which a hospital contributes to the health of its community elude measurement of the magnitude of an activity, the effect on health status (which is itself a vague term to measure), or the ability to attribute change directly and exclusively to the hospital's actions. (3) No clarity exists on “how much” a hospital should do for its community, either as a moral or regulatory obligation, or to achieve a change in the community's health status.

Although hospitals are able to play an important role in filling a gap of access and coverage for some individuals, forcing a hospital to engage in random activities with unclear amounts of resources is not a sufficient policy approach to improve health and wellbeing of a community or population. Community benefit laws and regulations, such as the ones currently on the books in the USA, might encourage community engagement. However, the lack of clear guidelines, arbitrary measures of process and outcomes, and no definitive targets to assess hospitals' contributions to community health result in these laws and regulations being costly but not necessarily effective. Are we asking hospitals/health systems to do more than they were designed to do? A thoughtful and thorough policy analysis of the community benefit policy in effect in the USA, potentially comparing states that do and do not require specific dollar amounts of attributed activities, would contribute to assessing the strengths and weaknesses of the current community benefit regulations.

To promote hospitals' contributions to their communities, we recommend that governmental policies of the future seek evidence-based, effective, measurable ways to involve organizations in improving the health of communities, allowing hospitals first and foremost to care for patients, and rewarding rather than penalizing engagement in activities aimed at improving the health of their communities more broadly.

## Author contributions

CC, CE, SS, and JT contributed to the conceptualization, writing, and editing of this manuscript. All authors contributed to the article and approved the submitted version.

## Conflict of interest

The authors declare that the research was conducted in the absence of any commercial or financial relationships that could be construed as a potential conflict of interest.

## Publisher's note

All claims expressed in this article are solely those of the authors and do not necessarily represent those of their affiliated organizations, or those of the publisher, the editors and the reviewers. Any product that may be evaluated in this article, or claim that may be made by its manufacturer, is not guaranteed or endorsed by the publisher.
